# Artificial intelligence–based quantitative computed tomography assessment of shoulder muscle and its association with outcomes after reverse total shoulder arthroplasty

**DOI:** 10.1016/j.jseint.2026.101753

**Published:** 2026-06-26

**Authors:** Sadaki Mitsuzawa, Akira Fujita, Hisataka Takeuchi, Koji Sakagami, Tadashi Yasuda, Shuichi Matsuda

**Affiliations:** a Department of Orthopaedic Surgery, Kyoto University Graduate School of Medicine, Kyoto, Japan; b Department of Orthopaedic Surgery, Kobe City Medical Center General Hospital, Kobe, Japan

**Keywords:** Muscle segmentation, AI, Rotator cuff, Fatty infiltration, Computed tomography, Reverse shoulder arthroplasty

## Abstract

**Background:**

Muscle atrophy and fatty infiltration of the shoulder muscles are known to influence clinical outcomes after reverse total shoulder arthroplasty (rTSA). Although quantitative evaluation of rotator cuff muscles has been increasingly reported, most previous studies have focused on intrinsic muscles and relied on two-dimensional or manual assessment methods. The purpose of this study was to evaluate shoulder muscle volume and composition using an artificial intelligence–based automated segmentation method on pre-operative computed tomography (CT) images and to investigate their associations with post-operative range of motion (ROM) after rTSA.

**Methods:**

This retrospective study included 13 patients (14 shoulders) who underwent rTSA for cuff tear arthropathy and related conditions with a minimum post-operative follow-up of 1 year. Pre-operative CT scans with 1-mm slice thickness were obtained within four weeks before surgery. Shoulder muscles, including intrinsic and extrinsic muscles, were automatically segmented using a deep learning–based model. Muscle volume and composition—functional muscle (FM), low-attenuation muscle, and adipose tissue (AT)—were quantified and normalized to scapular bone volume. Associations between muscle parameters and post-operative active ROM were analyzed using Spearman correlation coefficients.

**Results:**

The supraspinatus (SSP) exhibited a higher proportion of AT, possibly reflecting fatty infiltration, and was the only muscle showing a relatively balanced distribution of FM, low-attenuation muscle, and AT. The proportion of FM in the SSP was positively associated with active anterior elevation (AE), whereas the proportion of AT in the SSP and subscapularis (SSc) was negatively associated with active AE. In addition, total deltoid muscle volume and FM volume of the SSP were positively associated with post-operative AE. The AT volume of the SSc were negatively associated with active AE. No significant associations were identified between muscle composition or volume and post-operative external rotation.

**Conclusion:**

Artificial intelligence–based automated segmentation on pre-operative CT enabled detailed quantitative assessment of shoulder muscle volume and composition. Post-operative ROM tended to be associated with the deltoid, SSP, and SSc. Although these findings should be interpreted with caution due to limited statistical power, this exploratory feasibility study provides preliminary insights and may serve as a basis for future investigations.

Rotator cuff tear is a common condition in the elderly population. Including asymptomatic cases, the overall prevalence of rotator cuff tears is reported to be 62% in patients older than 80 years.^15^ It is well established that muscle atrophy and fatty infiltration of the rotator cuff muscles significantly affect shoulder functional outcomes following both arthroscopic repair and arthroplasty.^2^^,^^11^^,^^19^

The most widely accepted method for evaluating fatty infiltration is the Goutallier classification.^6^ Despite its simplicity, reproducibility, and widespread use, this classification system has inherent limitations, including substantial interobserver variability due to the subjective and visual nature of the assessment.^14^ Recent studies have described quantitative methods for assessing fatty infiltration. Furthermore, evaluation techniques have evolved from two-dimensional cross-sectional area measurements to three-dimensional (3D) volumetric analyses and from manual segmentation to deep learning–based automated segmentation.^6^^,^^10^ However, even in recent large-scale deep learning–based automated 3D assessment study, analyses have largely been limited to intrinsic rotator cuff muscles, whereas extrinsic muscles, such as the deltoid and trapezius, have not been evaluated.^10^ The volume and quality of both intrinsic and extrinsic shoulder muscles may be important factors influencing post-operative clinical outcomes after arthroplasty; however, these associations have rarely been investigated.

The purpose of this study was to determine whether shoulder muscles can be accurately segmented and reliably evaluated using an artificial intelligence (AI)-based automated method on pre-operative computed tomography (CT) images and to investigate the associations between muscle volume and quality and post-operative outcomes following reverse total shoulder arthroplasty (rTSA). If these associations are clearly established, post-operative outcomes may be predicted based on pre-operative muscle volume and composition, potentially serving as a useful reference for surgical decision-making in the future. As an initial step, this study is positioned as an exploratory investigation and a feasibility study.

## Materials and methods

This study was performed in accordance with the principles of the Declaration of Helsinki (as revised in 2013) and approved by the ethics committee of our institution (approval no. 25229). Informed consent was obtained from all patients before their inclusion in the study and after publication of the anonymized results.

This study was designed as a retrospective analysis of patients with cuff tear arthropathy (CTA) who underwent rTSA at our institution between January 2022 and December 2024. The inclusion criteria were patients with CTA who were judged to be suitable candidates for rTSA by 2 shoulder surgeons and who had a minimum post-operative follow-up of 1 year. Patients younger than 20 years of age, those with fractures, and those with insufficient imaging data were excluded.

All patients underwent a standardized pre-operative imaging protocol. Within four weeks prior to rTSA, CT was performed with a slice thickness of 1 mm to ensure complete visualization of the scapula.

**Figure 1:**
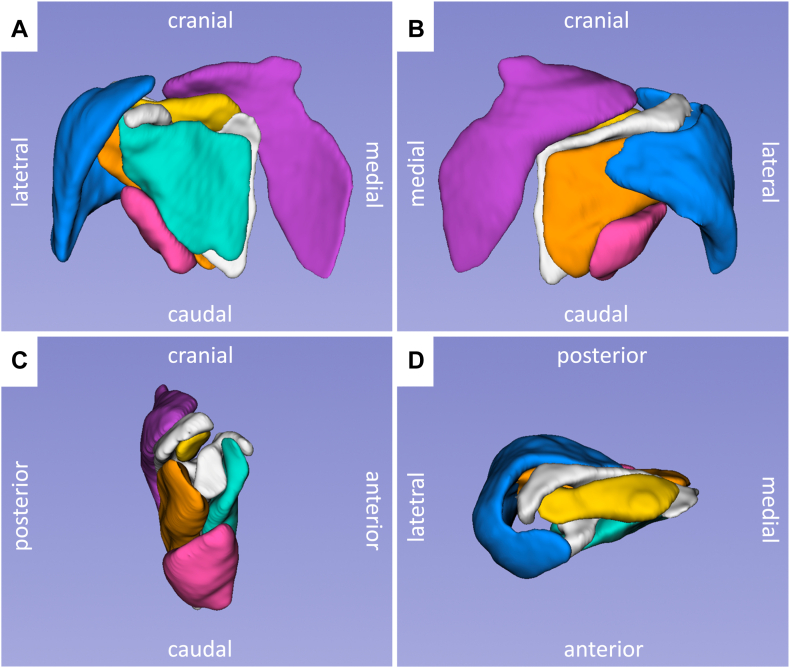
Three-dimensional reconstruction images of the shoulder muscles and scapular bone. *Blue*: deltoid; purple: trapezius; *green*: subscapularis (SSc); *yellow*: supraspinatus (SSP); *orange*: infraspinatus and teres minor (ISPTm); *pink*: teres major (TM). (**A**) Anterior view; (**B**) posterior view; (**C**) lateral view (deltoid removed); (**D**) cranial view (trapezius removed).

The scapula and surrounding shoulder muscles were automatically segmented using “TotalSegmentator,” a validated deep learning model.^16^ The deltoid, trapezius, subscapularis (SSc), supraspinatus (SSP), infraspinatus and teres minor (ISPTm), and teres major (TM) were identified (Fig. 1). As a quality check, all segmentations were visually inspected, and no obvious errors were identified; therefore, no manual corrections were required.

**Figure 2:**
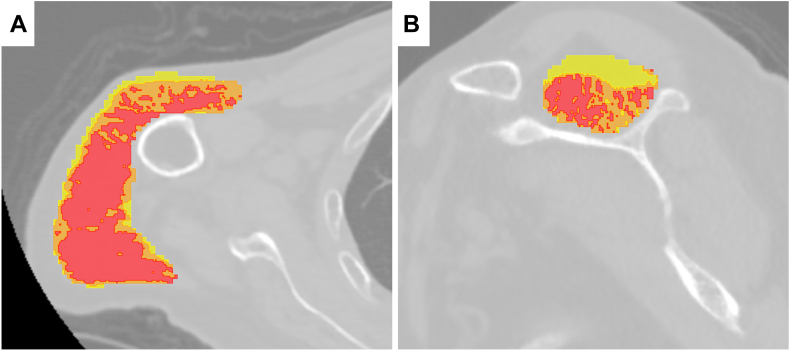
Distribution of muscle composition within each muscle. *Red*: functional muscle (FM); *orange*: low-attenuation muscle (LAM); *yellow*: adipose tissue (AT). (**A**) Deltoid in an axial slice; (**B**) supraspinatus (SSP) in a sagittal slice.

Following automatic segmentation, muscle phenotyping was performed for each of the 6 muscles. The regions of interest were automatically divided into 3 components based on radiodensity, as previously reported by Goodpaster et al^5^ functional muscle (FM; 30–150 HU), low-attenuation muscle (LAM; −30 to 30 HU), and adipose tissue (AT; −190 to −30 HU). The proportion of each component was calculated for each muscle (Fig. 2).

Total muscle volume and the volumes of each component (FM, LAM, and AT) were normalized by dividing the target volume by the volume of the scapular bone.^17^ This normalization was intended to reduce the influence of interindividual differences in body size on muscle volume measurements.

Data are presented as means and standard deviations. Differences in muscle composition (FM, LAM, and AT) were analyzed using the Tukey–Kramer method. Spearman correlation coefficients (ρ) were calculated to assess associations between muscle composition or volume and post-operative active range of motion (ROM). Correlation coefficients of 0.20–0.39, 0.40–0.59, and 0.60–1.0 were interpreted as mild, moderate, and strong correlations, respectively. Confidence intervals (CIs) for the correlation coefficients were estimated using a nonparametric bootstrap method with 2,000 resamples. Given the exploratory nature of this study and the small sample size, a post hoc power analysis was performed for the variable pair demonstrating the highest Spearman ρ. All statistical analyses were performed using SPSS (version 25; SPSS Corp., Armonk, NY, USA), and statistical significance was set at *P* < .05. To assess internal reliability, the same CD-ROM dataset was reanalyzed 6 months later using the identical workflow for automatic segmentation and muscle phenotyping, and all measurements were found to be identical to those obtained in the initial analysis.

## Results

During the study period, 13 patients (14 shoulders) were included. Patient demographics are summarized in Table I. The cohort consisted of 3 men and 10 women, with a mean age of 78.9 years. The mean height and body weight were 152.6 cm and 59.0 kg, respectively. The most common indication for rTSA was CTA (10 shoulders), followed by osteonecrosis (3 shoulders) and rheumatoid arthritis (1 shoulder). At the final follow-up, the mean active ROM was 109.3° for anterior elevation (AE) and 29.3° for external rotation (ER).

**Table I:** Patient demographics.

Variable	13 patients 14 shoulder
Mean age (years)	78.9 ± 7.9
Male: female	3 : 10
Height (cm)	152.6 ± 11.5
Body weight (kg)	59.0 ± 18.1
Right: left	8 : 6
CTA: ON: RA	10 : 3: 1
Active ROM (anterior elevation)	109.3 ± 28.7
Active ROM (external rotation)	29.3 ± 20.6

*CTA*, cuff tear arthropathy; *ON*, osteonecrosis; *RA*, rheumatoid arthritis; *ROM*, range of motion.

The volume and composition of each muscle are summarized in Table II. The total volume of the deltoid muscle was 201.7 ± 66.1 cm^3^, making it the largest muscle among the 6 evaluated. The trapezius, SSc, and ISPTm each had volumes of approximately 100 cm^3^. In contrast, the volumes of the SSP and TM were less than 40 cm^3^, which was smaller than those of the other muscles. The mean volume of the scapular bone was 83.5 ± 26.1 cm^3^.

**Table II:** Volume of each shoulder muscle and the scapular bone.

Muscle	Total volume	Functional muscle	Low-attenuation muscle	Adipose tissue
Deltoid	201.7 ± 66.1	123.3 ± 55.7	56.0 ± 15.2	22.4 ± 8.6
Trapezius	110.3 ± 47.9	59.3 ± 28.3	32.4 ± 13.9	18.7 ± 9.2
SSc	100.9 ± 35.6	56.3 ± 27.3	33.6 ± 10.3	11.0 ± 7.6
SSP	35.7 ± 10.3	14.5 ± 8.4	11.7 ± 3.9	9.5 ± 3.8
ISPTm	108.5 ± 33.2	57.2 ± 25.3	34.9 ± 11.8	16.3 ± 9.4
TM	38.1 ± 12.7	24.3 ± 9.9	11.0 ± 4.5	2.9 ± 3.4
Scapular bone	83.5 ± 26.1			

*SSc*, subscapularis; *SSP*, supraspinatus; *ISPTm*, infraspinatus and teres minor; *TM*, teres major.

**Figure 3:**
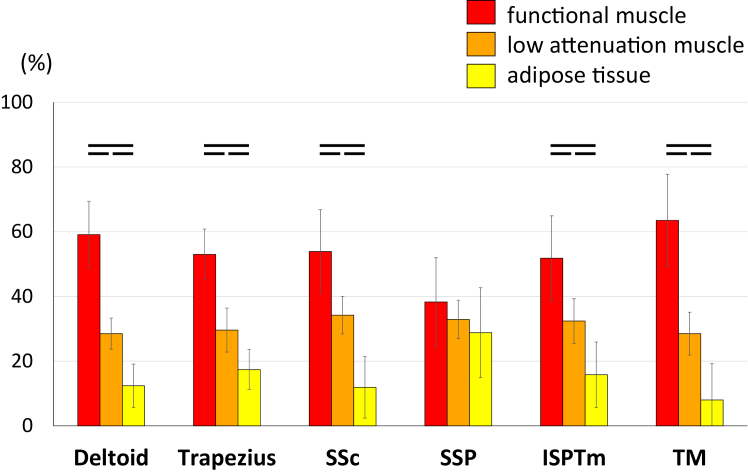
Percentage distribution of muscle composition in each muscle. Horizontal black lines above the bar graphs indicate statistically significant differences between pairs of muscle components. Among the evaluated muscles, the SSP exhibited a higher proportion of AT, and was the only muscle showing a relatively balanced distribution of FM, LAM, and AT. *FM*, functional muscle; *LAM*, low-attenuation muscle; *AT*, adipose tissue; *SSP*, supraspinatus; *SSc*, subscapularis; *ISPTm*, infraspinatus and teres minor; *TM*, teres major.

The muscle composition—FM, LAM, and AT—of each muscle is illustrated as percentages in Figure 3. The SSP exhibited a higher proportion of AT, possibly reflecting fatty infiltration, and was the only muscle showing a relatively balanced distribution of FM, LAM, and AT. Associations between muscle composition and active ROM are presented in Table III. The percentage of FM in the SSP was positively associated with active AE (ρ = 0.536, 95% CI = 0.03 ∼0.82, *P* = .048). Conversely, the percentage of AT in the SSc and SSP was negatively associated with active AE (ρ = −0.549, 95% CI = −0.91 ∼ 0.02, *P* = .042 and ρ = −0.547, 95% CI = −0.87 ∼ 0.02, *P* = .043, respectively). Associations between muscle composition and active ER were limited.

**Table III:** Correlation between the composition of each muscle and post-operative range of motion.

Muscle	Composition (%)	Anterior elevation			External rotation		
		ρ	95% CI	*P* value	ρ	95% CI	*P* value
Deltoid	FM	0.219	−0.44 ∼ 0.77	.451	0.184	−0.51 ∼ 0.71	.528
	LAM	−0.303	−0.71 ∼ 0.35	.292	−0.244	−0.77 ∼ 0.41	.400
	AT	−0.248	−0.78 ∼ 0.40	.392	−0.096	−0.65 ∼ 0.53	.745
Trapezius	FM	0.226	−0.45 ∼ 0.82	.437	0.127	−0.57 ∼ 0.74	.666
	LAM	0.104	−0.56 ∼ 0.81	.723	−0.200	−0.74 ∼ 0.44	.493
	AT	−0.470	−0.92 ∼ 0.13	.090	0.131	−0.53 ∼ 0.74	.655
SSc	FM	0.385	−0.30 ∼ 0.91	.174	0.240	−0.39 ∼ 0.76	.409
	LAM	0.075	−0.62 ∼ 0.79	.798	−0.107	−0.64 ∼ 0.49	.717
	AT	−0.549	−0.91 ∼ 0.02	.042	−0.313	−0.80 ∼ 0.28	.275
SSP	FM	0.536	0.03 ∼ 0.82	.048	0.184	−0.49 ∼ 0.84	.528
	LAM	0.153	−0.43 ∼ 0.67	.602	−0.076	−0.66 ∼ 0.50	.797
	AT	−0.547	−0.87 ∼ 0.02	.043	−0.216	−0.83 ∼ 0.46	.459
ISPTm	FM	0.310	−0.26 ∼ 0.74	.281	0.187	−0.47 ∼ 0.80	.523
	LAM	0.137	−0.45 ∼ 0.66	.640	0.071	−0.53 ∼ 0.59	.809
	AT	−0.432	−0.82 ∼ 0.21	.123	−0.224	−0.87 ∼ 0.44	.440
TM	FM	0.135	−0.44 ∼ 0.68	.645	−0.060	−0.73 ∼ 0.59	.839
	LAM	0.066	−0.56 ∼ 0.59	.821	0.229	−0.45 ∼ 0.82	.431
	AT	−0.142	−0.72 ∼ 0.48	.629	−0.224	−0.70 ∼ 0.41	.440

*SSc*, subscapularis; *SSP*, supraspinatus; *ISPTm*, infraspinatus and teres minor; *TM*, teres major; *FM*, functional muscle; *LAM*, low-attenuation muscle; *AT*, adipose tissue; *CI*, confidence interval.

**Figure 4:**
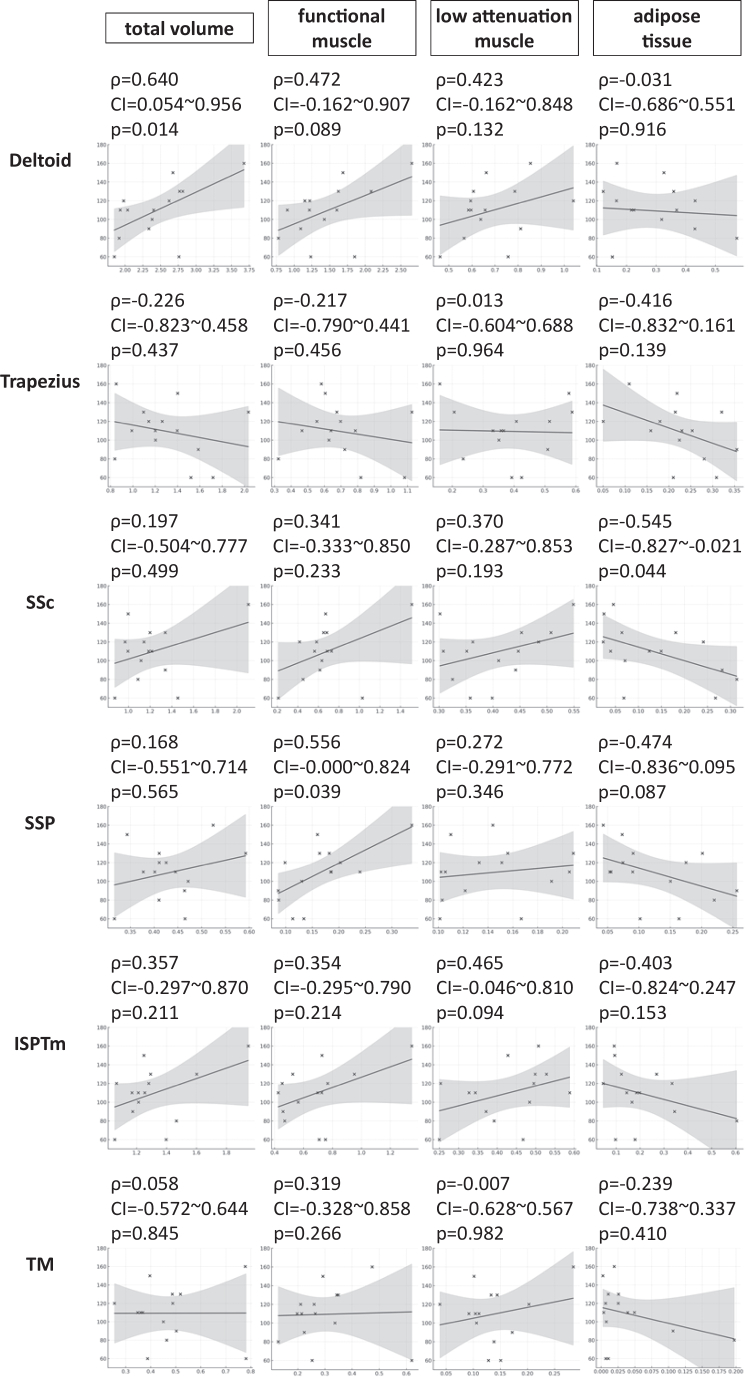
Spearman correlation analysis between anterior elevation (AE) and muscle volume. The total volume of the deltoid and the FM volume of the SSP were positively associated with active AE. The AT volume of the SSc were negatively associated with active AE. *FM*, functional muscle; *AT*, adipose tissue; *SSP*, supraspinatus; *SSc*, subscapularis.

**Figure 5:**
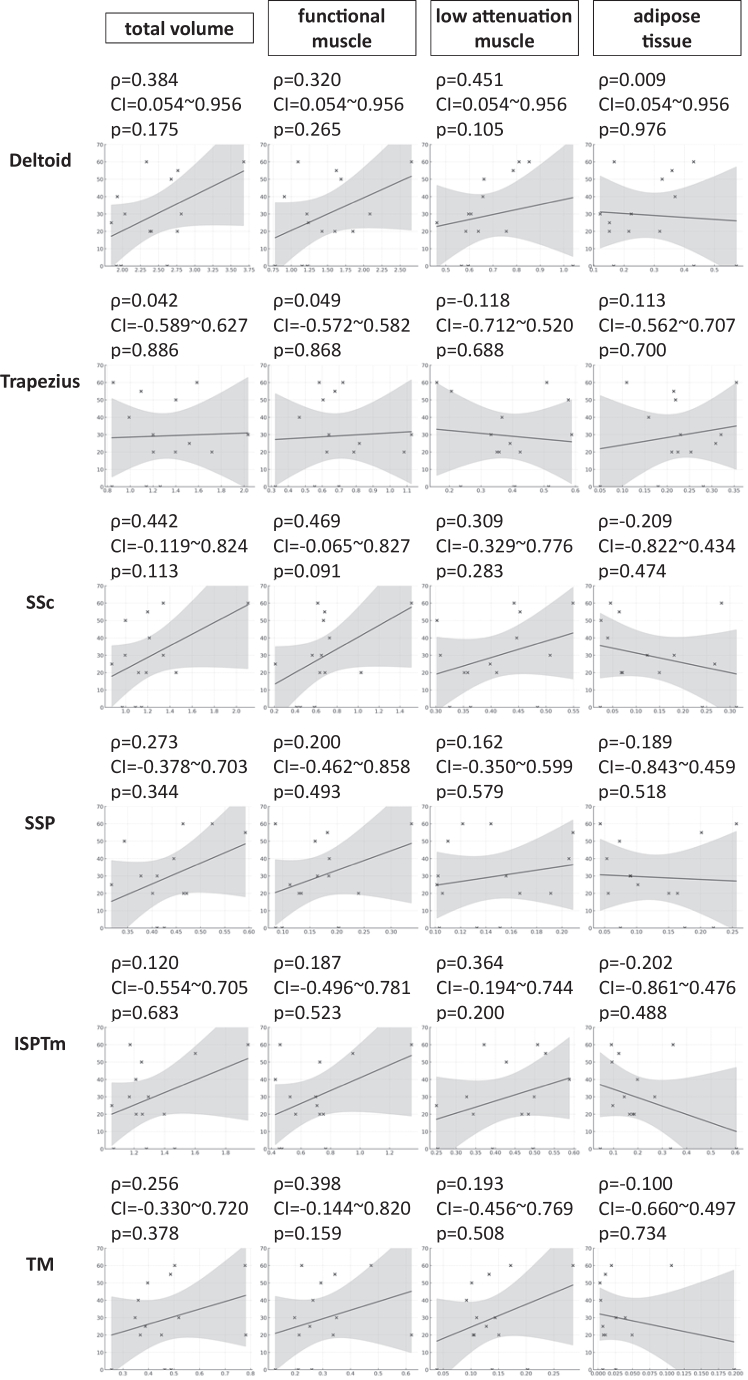
Spearman correlation analysis between external rotation (ER) and muscle volume. The LAM volume of the deltoid, as well as the total and FM volume of the SSc, tended to be positively associated with active ER; however, these associations did not reach statistical significance. *FM*, functional muscle; *LAM*, low-attenuation muscle; *SSc*, subscapularis.

Associations between muscle volume and active ROM are shown in scatter plots with regression lines and 95% CIs (Figs. 4 and 5). All muscle volume measurements were normalized to the volume of the scapular bone. The total volume of the deltoid and the FM volume of the SSP were positively associated with active AE (ρ = 0.640, 95% CI = 0.054 ∼ 0.956, *P* = .014 and ρ = 0.556, 95% CI = −0.000 ∼ 0.824, *P* = .039, respectively). The AT volume of the SSc were negatively associated with active AE (ρ = −0.545, 95% CI = −0.827 ∼ −0.021, *P* = .044). The LAM volume of the deltoid, as well as the total and FM volume of the SSc, tended to be positively associated with active ER; however, these associations did not reach statistical significance.

The post hoc power for detecting the observed correlation coefficient of r = 0.640 (total volume of the deltoid and active AE) with a sample size of 14 at a two-sided alpha level of 0.05 was 0.755.

## Discussion

In this patient cohort, shoulder muscles could be accurately segmented using an AI-based automated method on pre-operative CT images, and quantitative data were available for assessment. Among the 6 evaluated muscles, only the SSP demonstrated a distinct muscle composition, with relatively converged proportions of FM, LAM, and AT. The proportion of FM in the SSP was positively associated with active AE, whereas the proportion of AT in both the SSc and SSP was negatively associated with active AE. In addition, the total volume of the deltoid and the FM volume of the SSP were positively associated with active AE.

There are 3 important factors that can strongly influence the evaluation of rotator cuff muscle atrophy and fatty infiltration. The first factor is the imaging modality. Fuchs et al^4^ investigated reproducibility using CT and magnetic resonance imaging (MRI) and reported that interobserver reproducibility for grading fatty degeneration was good to excellent for both modalities. However, the correlation between CT- and MRI-based assessments was only fair to moderate and remained unsatisfactory.^4^ MRI enables comprehensive evaluation of rotator cuff pathology, including tear configuration, muscle atrophy, and associated soft-tissue abnormalities, without exposure to ionizing radiation. In contrast, CT offers shorter acquisition times and allows quantitative assessment of fatty infiltration using muscle density measurements such as Hounsfield units, providing highly reproducible evaluations. However, CT has inherent limitations in evaluating fine soft-tissue structures, such as the intrinsic aponeurosis and detailed muscle architecture. These features may not be fully captured by CT-based segmentation, particularly near the tendon insertion, and MRI may be more suitable for their assessment.

The second factor is the dimensionality of assessment. Two-dimensional evaluation typically refers to a single-slice–based assessment using an image at the scapular Y-view.^4^ This approach is practical for pre-operative evaluation because it requires minimal time and effort and is therefore widely applicable in clinical settings. However, because only a limited portion of the muscle is assessed, this method is susceptible to variability related to slice selection and may fail to capture heterogeneous patterns of fatty infiltration within the muscle. Consequently, single-slice–based grading may not adequately represent whole-muscle fatty infiltration. To address these limitations, several recent studies have adopted 3D evaluation approaches based on whole-muscle segmentation, allowing comprehensive and quantitative assessment of fatty infiltration across the entire muscle volume.^10^^,^^17^ This methodology provides continuous variables rather than ordinal grades, reduces observer dependency, and improves reproducibility.

The third factor is the method of segmentation. Manual segmentation is generally considered the gold standard because of its high anatomical fidelity, particularly when performed by experienced operators.^3^ However, it requires slice-by-slice delineation and is time-consuming, labor-intensive, and subject to observer dependency.^17^ In contrast, AI-based segmentation techniques have rapidly evolved, enabling efficient and highly reproducible assessments and representing a promising approach for large-scale studies and future clinical implementation.^10^^,^^16^ In the present study, no obvious segmentation errors requiring manual correction were identified, and repeated analysis using the same dataset yielded identical measurements, supporting the reproducibility of the automated approach. Nevertheless, we did not perform a direct comparison with manual segmentation and therefore cannot fully assess relative accuracy. Future studies directly comparing manual and automated techniques are warranted.

The concept of the present study was AI-based automated 3D segmentation using CT. In previous studies, the target muscles were generally limited to intrinsic muscles, such as the 4 rotator cuff muscles.^10^^,^^17^ To our knowledge, the present study is the first to investigate extrinsic muscles—including the deltoid, trapezius, and TM—in addition to intrinsic shoulder muscles, all of which were accurately segmented and reliably evaluated on pre-operative CT images. The volumes of the SSc, SSP, ISPTm, TM, and scapular bone were similar to those reported in previous studies.^7^^,^^17^ Although the volumes of the deltoid and trapezius muscles were slightly lower than those reported in the literature, they were considered to be within the expected range, given that the study cohort predominantly consisted of elderly female patients with lower body size.^1^^,^^9^^,^^12^

In rTSA, the deltoid muscle is intended to compensate for deficient rotator cuff function, and the anterior and middle portions of the deltoid are particularly associated with forward elevation. Post-operative ROM may be determined by both the direction of force generation (eg, pennation angle, defined as the angle between the muscle fibers and the line of pull) and the magnitude of muscle force. Muscle force itself is influenced by muscle volume and quality, including fatty infiltration, which affect the contractile capacity of the muscle.^8^ Previous studies have suggested that pre-operative deltoid muscle volume may be associated with post-operative ROM, whereas muscle quality, such as fatty infiltration, shows weaker or inconsistent associations with post-operative motion.^9^^,^^19^ Consistent with these findings, our study demonstrated that deltoid muscle volume was significantly associated with post-operative forward elevation, whereas deltoid muscle composition was not. Extrinsic muscles, such as the trapezius and TM, have been underexplored in rTSA. We hypothesized that these muscles might contribute to post-operative ROM; however, no meaningful associations were observed. These findings suggest that extrinsic muscles other than the deltoid may play a limited role, supporting a clinical focus on the deltoid, including pre-operative rehabilitation strategies.

Rhee et al^13^ reported that lower degrees of pre-operative fatty infiltration of the SSc, reflecting better muscle quality, were associated with improved post-operative functional internal rotation. However, other studies, including systematic reviews, have suggested that the association between pre-operative SSc status and post-operative ROM is limited or inconsistent. In the present study, the proportion of AT within the SSc was negatively associated with active AE. Although we initially hypothesized that SSc composition would be associated with poorer ER, no such association was observed.^11^^,^^13^^,^^19^ The SSc tendon is routinely detached and subsequently repaired during the surgical approach. The degree of tensioning and healing varies among cases, which may substantially influence post-operative ER. In addition, the altered biomechanics after rTSA, including medialization/lateralization of the center of rotation and humeral lengthening, change ISPTm muscle length and moment arms. These factors may partly explain the lack of association with post-operative ER.

Few studies have clearly demonstrated an association between the pre-operative status of the SSP and post-operative ROM after rTSA for glenohumeral osteoarthritis. However, a systematic review reported that fatty infiltration of the SSP is associated with poorer post-operative ROM.^11^ In fracture settings, healing of the greater tuberosity—where the SSP inserts—has also been recognized as an important factor influencing post-operative outcomes. In the present study, both the FM volume and muscle composition of the SSP were associated with post-operative active AE. From a biomechanical perspective, the SSP may contribute to glenohumeral joint stability by providing a compressive force that counteracts superior translation and facilitates efficient deltoid function during arm elevation. In addition, preservation of SSP integrity may help maintain cuff tension and coordinated scapulohumeral motion. These mechanisms may partly explain the observed association.^18^^,^^19^

Several limitations should be acknowledged. First, the relatively small sample size may have reduced statistical power and limited the generalizability of the findings. Although the achieved maximum power approached 0.755 for the correlation, it did not reach the conventional 0.80 threshold. Given the exploratory design, small sample size, and multiple correlations performed, there is a potential risk of type I error. Therefore, the findings should be interpreted with caution. The second limitation relates to the patient cohort, including potential selection bias from a single institution, short follow-up duration, and the use of ROM alone without patient-reported outcomes. Although normalization to scapular bone volume was performed, no adjustment for confounding factors (eg, age, sex, indication, and baseline ROM) was made, and multivariable analysis was not feasible. As this was an exploratory feasibility study, these limitations warrant further investigation in larger, more comprehensive studies. Third, muscle segmentation and evaluation of fatty infiltration were performed using an AI-based automated approach on thin-slice CT images. Although AI-based segmentation enables rapid and reproducible analysis, its accuracy around the shoulder girdle may be affected by complex anatomy and pathological changes, potentially introducing measurement variability. Nevertheless, the muscle volume and composition values obtained in this study were largely comparable to those reported in previous literature, suggesting that the measurements fall within an acceptable and expected range.

## Conclusion

Using an AI-based automated approach, shoulder muscles were successfully segmented on pre-operative CT, enabling detailed quantitative evaluation of muscle volume and composition. Post-operative ROM showed a trend toward association with the deltoid, SSP, and SSc; however, given the limited statistical power, these findings should be interpreted with caution. With a larger sample size and appropriate adjustment for confounding factors, AI-based quantitative assessment of shoulder muscles may help determine whether pre-operative evaluation can be used to inform surgical decision-making in patients undergoing rTSA.

## Disclaimers:

Funding: No funding was disclosed by the authors.

Conflicts of interest: The authors, their immediate family, and any research foundation with which they are affiliated have not received any financial payments or other benefits from any commercial entity related to the subject of this article.

## Availability of data

The datasets used and/or analyzed during the current study are available from the corresponding author upon reasonable request.
